# Conflicting trends in violent crime measured by police recorded crime and the crime survey in England and Wales since 2010

**DOI:** 10.1371/journal.pone.0324272

**Published:** 2025-06-04

**Authors:** Brian Joseph Francis, Sylvia Walby

**Affiliations:** 1 School of Mathematical Sciences, Lancaster University, Lancaster, United Kingdom; 2 Department of Law and Criminology, Royal Holloway, University of London, London, United Kingdom.; Utrecht University: Universiteit Utrecht, NETHERLANDS, KINGDOM OF THE

## Abstract

Police recorded violent crime (PRC) and the Crime Survey of England and Wales (CSEW) show substantially different trends in the rates of violent crime according to the Office of National Statistics (ONS), with rates rising in police data and falling in survey data. Both the PRC and CSEW have suffered periods in which the UK Statistics Authority has withdrawn their quality approval as ‘national statistics’. This paper investigates a possible seven reasons for the disparity in the trend and volume of violent crime between the PRC and CSEW, with a focus on the processes of measurement deployed. The paper offers a new way to compare the methods and outcomes of the two data sources, by developing an ‘aligned’ data set to support comparison of trends in the PRC CSEW data since 2010. It analyses data from the PRC and from different sections of the CSEW, the main face-to-face module, the self-completion module on domestic abuse, and the children’s module asked of those aged 10–15 years. We offer new estimates of the volume and discussion on the trend in violent crime since 2010. We estimate that there were 5,164,983 violent crimes in 2022/3. This is significantly higher than the estimate provided by the ONS based on CSEW data. The estimate of the trend is uncertain, but challenges over-confidence in the assumption that it is declining. We conclude that improvements in police accuracy in recording crime explains part of the difference, and the exclusion from sampling of vulnerable groups by the CSEW another part, with the recent reduction in the survey’s response rate to 42% giving further quality concerns. We determine that the CSEW has always underestimated violent crime, and this has become visible now that police data has improved.

## Introduction

Violent crime is going down, according to the Crime Survey for England and Wales (CSEW). Violent crime is going up, according to police recorded crime statistics (PRC). Historically, the crime survey (CSEW) has identified more violent crime than the police (PRC), but this changed in 2017, and in 2023, the police recorded more than twice as many violent crimes as the survey.

Both data sources have problems and have, for specific periods of time, lost the quality kitemark of being “accredited official statistics” as recognised by the UK Government’s Office for Statistics Regulation (OSR). Police recorded crime data in England and Wales has long been criticised for its inconsistency and underreporting of crimes. In 2014, its designation as a “national statistic” was removed by the UK Statistics Authority, and the HMIC led efforts to improve its quality of police data. The ONS has long treated the CSEW as the better source of estimates of the trend and volume of violent crimes, but the CSEW also lost its quality designation in 2022 for a period of time amid concern for low response rates.

Both data sources insufficiently address the dark figure of crime, as police data omits violence that is not reported to or recorded by them, and the CSEW omits those outside their sampling frame and those inside the sampling frame who do not respond or disclose.

Currently, the gold standard for violent crime reporting in England and Wales is the CSEW. Thus, a recent Office for Statistics Regulation report on the quality of police recorded crime statistics [1] lists the CSEW as the preferred source for violent crime. Academics, in general, have taken this view. Thus, Davies and Farrell [2] recently wrote that

*As of 2024, violence, burglary and car crime have been declining for 30 years and by close to 90%, according to the Crime Survey for England and Wales) – our best indicator of true crime levels. Unlike police data, the CSEW is not subject to variations in reporting and recording*.

There have been numerous criticisms of both data sources. The CSEW has been criticised for capping high frequency victimisations at a low value, where the same or similar violent events occur repeatedly to a victim [3–5], meaning that only the first few of these victimisations make it into official statistics. It has also been criticised for its restricted definition of violence [6], and for prioritising property crimes above violence in determining how to categorise a crime with multiple components [7]. Researchers have also reported that the CSEW excludes the most vulnerable from its sampling frame [8].

Criticisms of police recorded crime (PRC) are often concerned about the accuracy of and variability in the way police forces interpret the counting rules. PRC, historically, reported smaller numbers, while the CSEW reported larger numbers that included both police recorded crime and crimes not reported to the police. Recently, an academic assessment of the quality of police data and the CSEW found that in relation to variations between small areas, police data was more consistent over time than estimates from the crime survey [9].

There have been previous discussions of the methodological issues involved in using police and survey data to analyse changes in violent crime [10,11]. Probably the most extensive comparison of rates of violence in police recorded crime and the CSEW was by Ariel and Bland [12]. Examining data up to 2018, they found that police recorded crime was increasing while CSEW violence was decreasing, but that police violent crime was still less than survey violent crime. They suggested that that “the mechanism at play in the figures is a genuine rise in crime (not just the reporting or recording of it)”.

Our analysis reported here extends the data series up to 2023. We now find more violent crime in the police data than in the survey data. We show that in 2017/8, the police started to record more violent crime than the survey, and in 2023, the police recorded over twice as many violent crimes as the survey. By combining knowledge of the extent of reporting violent crime to the police from the survey, we produce a new estimate of the volume of violent crime, which is more than five times the official estimate from the CSEW.

The resolution of the methodological debates is important for the improvement of the data needed to test theory. While there are many accounts suggesting a long-run drop in violent crime [13,14], there are exceptions[5], and its explanation remains contested [15]. There are long-standing theoretical debates as to the relative importance of different causes of variations in rates of violence, including social institutions generating self-control [16], responses by the state and police [17], levels of security [18], and variations in inequality[19–21].

We identify seven methodological reasons that might explain why the amount and trends in violent crime differ between the survey and the police. We assess them, using logic, literature, and data. We offer an original investigation of the data provided by the CSEW and PRC on the extent and rate of change in violent crime, disaggregating by severity of violent crime.

The seven possible reasons we address are:

1. There are differences between the survey (CSEW) and police (PRC) because they use different temporal units.
2. There are differences in the definition of the violent crime between the police and the survey, including which violent crimes are included, and different rules on counting and capping violent crimes when there is not one victim, one crime event, and one reporting event.
3. There are differences between the survey and police because they refer to different populations by design, including children, and those not living in settled residential households.
4. Violent crime reported in the CSEW is declining because the response rate of the survey is declining and increasingly omitting the most marginalised and minoritised.
5. Violent crime is differently disclosed to the CSEW, including to its main module and self-completion module.
6. Violent crime reported by the police is increasing because more people who experience violent crime are reporting or having it reported to the police
7. Violent crime recorded by the police is increasing because the police are getting better at recording the violent crime reported to them.

The methodology of what and how to report in official publications matters because it affects substantive findings on the extent, distribution, and trend in violent crime, which have implications for theories of violence, crime, and inequality and for policies on official statistics and on violent crime.

We conclude that violent crime has always been under-estimated by the CSEW because of the sampling frame and its implementation, the methodology used, and questions asked. This is becoming visible as the quality of police data is improving.

This paper proceeds as follows. In the next section, we discuss the various quality assessments of these two data sources, the different concepts of the dark figure of crime, and introduce the two sets of data. The following section examines the seven possible reasons that have been postulated to explain the differences between police and survey data, offering an initial assessment using literature and logic. The next section discusses the methodology used by the paper to further address these issues, including creating an aligned subset of data to assist making comparisons. Then we offer an analysis of trends using this approach. and a new estimate of the amount of violent crime. The final sections discuss the seven possible explanations of the differences and draw conclusions.

### Quality assessments

In England and Wales, the gold standard for measuring crime incidents is currently recognised as being the Crime Survey for England and Wales (CSEW). For example, in December 2023, the Office for National Statistics (ONS) stated that

*The CSEW is a better indicator of long-term trends for the crime types and population it covers than police recorded crime because it is unaffected by changes in levels of reporting to the police or police recording practices*. [22].

Official views on police recorded crime data, however, have changed over time. Since 2002, PRC data has been collected using a victim-based approach, counting one crime per victim report, and this allows recorded crime to be compared with population crime estimates from CSEW. Up to 2014, PRC and CSEW data were published alongside each other, to “provide a fuller picture than was possible for either series alone” [23]). However, in the 2010s there was increasing government, academic, and public concern about the quality of police crime figures [24]. For example, the *Guardian* reported on an Her Majesty’s Inspectorate of Constabulary (HMIC) inspection of Kent Police, where one in ten crimes had not been reported correctly [25]. Similarly, *The Times* reported in an editorial that “Parliament is shocked by new evidence that police manipulate crime statistics” [26]. Early in 2014, the UK Statistics Authority (UKSA) removed the National Statistics designation from PRC data [27]. A review of the quality of police crime statistics in late 2014 [28] confirmed the lack of accuracy in the data, finding substantial under-recording. Overall, around 80% of crimes that should have been recorded were actually recorded, with even lower under-recording rates for sexual offences (74%) and violence (67%). Since that time, there have been substantial improvements in PRC data. A recent (2024) report on PRC statistics based on nine police forces concluded that “police forces are recording more accurately now than in 2014”, with a positive shift in the culture, and with 92.4% of crimes now accurately recorded [1]. There are however issues regarding consistency between police forces, which means that PRC has yet to recover National Statistics status. This meant that trends in crime since 2014 were reported by the ONS solely through crime survey (CSEW) estimates, and the two series were no longer reported side by side.

However, problems have also arisen with the CSEW, which was “temporarily” suspended from the National Statistic designation in 2022 at the request of the ONS [29], though it was restored by 2025. This was primarily due to the very low survey response rates in the recent surveys, following return to face-to-face interviews after the COVID pandemic. Yet, the ONS still considers the CSEW to be the preferred data source for measuring violent crime [1]. In the user guide, they discuss the divergence in the volume of crime in the two series, suggesting in 2023 that there were no obvious methodological changes to the survey that explained the increasing difference in the two series [30]. Yet, as the OSR [1] point out, in 2024 “*no crime statistics for England and Wales are published as accredited official statistics”*. This means that neither series (CSEW, PRC) has a continuous quality assurance in recent years. With both series compromised, the time is opportune to look again at both.

### The dark figure of crime

Underlying this discussion is the issue of the ‘dark figure of crime’. Neither of the two major data series is able to shed light on all crimes, for several reasons. [31] produces a typology of fifteen types of dark data, ranging from self-selection to data analysts do not even know are missing. The dark figure of crime refers to crime incidents which have taken place but have not been recorded either in administrative data or estimated in crime surveys.

**Fig 1:**
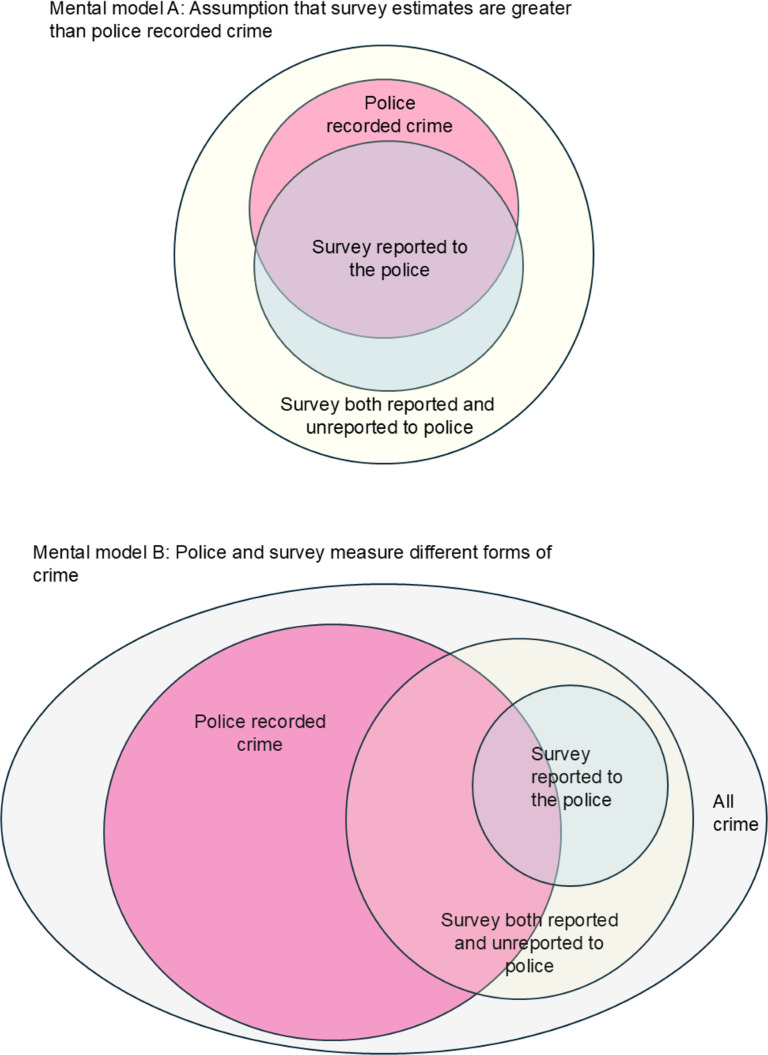
Mental models of the dark figure of crime.

The various meanings of the concept of the dark figure of crime used in criminology can be divided into two main models. Model A (see Fig 1) is a simple model, which assumes that police data is always going to under-record compared to crime surveys, since police data needs to be made known to and recorded by the police, whereas surveys are used to estimate both recorded and unrecorded crime.

In Model A, the dark figure can be estimated by examining the difference between the survey estimate (high) and police recorded data (low). This was the approach of Fohring [32] who examined the characteristics of those not reporting crime. Buil-Gil et al. [33] used crime survey estimates of crime together with the estimated proportion of crime reported to the police (also from the crime survey) to examine characteristics of non-reporters at the small area level. Recently, Wheeler and Piquero [34] used the US National Crime Victimization Survey to upscale police recorded domestic violence incidents at the local level. Neither of these studies considered the presence of dark data in the crime survey.

In Model B, there are more complex understandings of the dark figure of crime, in which all sources of crime data are assumed to have a dark figure element (see Fig 1). This is the approach taken by Biderman and Reiss. van Dijk and Riascos Villegas et al. [35–37], in which police data can provide some information on dark data in a crime survey, and crime survey data can provide some information on the dark figures in police data. If individuals can be linked across data sources, then it might be possible to estimate the dark figure by using multiple systems estimation [38], a method used in estimating the number of trafficked persons [39] and of drug users [40]. However, more commonly this approach is not possible since records on individuals cannot be linked since survey data is usually anonymised and police data usually be de-identified before it is released to researchers. The approach in Model B is that all forms of crime data have their dark component, and that there is no simple relationship between crime survey estimates and police recorded crime.

We start from the position of Model B, that there is a dark figure component in both police recorded crime data and crime survey estimates; and then investigate gaps in each data set and points of overlap between them in order to improve estimates.

### The two data sources: police and survey

There are two sets of data under discussion: police recorded crime (PRC), and the Crime Survey for England and Wales (CSEW).

#### Police recorded crime (PRC).

We refer to “*reported crime*” as crimes which have been reported to the police by the victim, friends of the victim, or others, and which is recorded by the police. The police may also record crime themselves without a member of the public being involved in its reporting. “*Recorded crime*” is a formal process where, since 2002, the police in England and Wales record crimes according to the National Crime Recording Standard, following counting rules [41].Not all crimes reported to the police are recorded – the offence needs to be a notifiable offence, it needs to be interpreted by the police as passing the threshold of being a crime, and it needs to be a crime against an identifiable victim or against society or the state. For many crime events, there is one crime for each victim, but in more complex circumstances, crimes are recorded according to Home Office counting rules [42]. This identifies which offence takes priority if two or more crimes are committed in the same crime event, and also how many crimes to record for a reported event. Published reports by ONS of police recorded crime used in this paper [43] define violence as including homicides, death or serious injury by unlawful driving, violence with injury, violence without injury, and stalking and harassment. We also used published data on the number of violent police recorded crimes flagged as domestic abuse [44].

#### Crime Survey for England and Wales (CSEW).

The (adult) CSEW (formerly the British Crime Survey) is a large, multi-stage survey carried out yearly in which a sample of respondents aged 16 or over resident in households in England and Wales are asked about their experiences as a victim of a range of crimes in the 12 months prior to the interview. The survey omits those in prison, care homes, domestic violence shelters and other residential accommodation from its sampling frame. As a victimisation survey, it measures crime not reported to the police as well as crimes reported to the police. The CSEW asks respondents if the crimes they are reporting to the survey were reported to the police. It currently has a sample size of around 35,000 achieved interviews per year. Supplied weights allow population estimates and rates to be produced from the sample counts. The number of crimes and victims for a variety of types of crime are measured through the face-to-face (F2F) victimisation module. Each potential crime incident is described in a free-text box recorded by the interviewer, and specialist coders determine whether the incident is in fact a crime, and what offence code to assign it to.

Domestic violence is measured in both the F2F and through a confidential computer-based module, which asks respondents to ‘self-complete’ (SC) a questionnaire about their experiences of abuse from partners, ex partners and the family in a manner that the interviewer cannot see. The CSEW definition of violence is more limited than police recorded crime. Fatal violence cannot be measured, and harassment, stalking, and coercion and control are excluded from the official estimates of CSEW violence in the main reports. While harassment, stalking, and coercion and control are included in estimates from the SC module, measurement of the *number of incidents* of domestic violence in the SC module is poor.

### Comparing the raw data from the police and survey

**Fig. 2:**
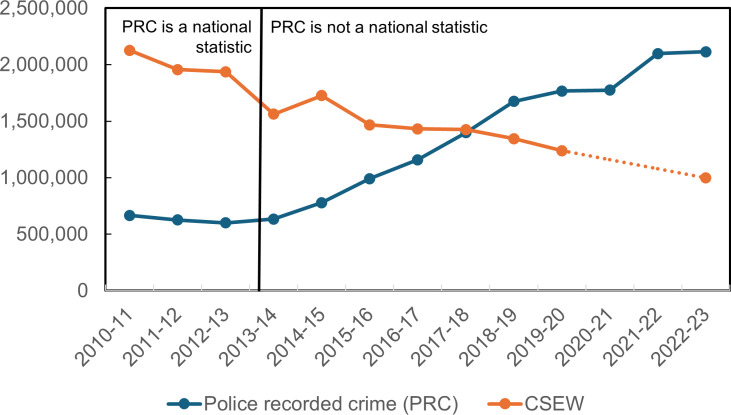
PRC and CSEW raw violent crimes in England and Wales, by year (financial year, April-March), 2010-2023.

Fig 2 shows the *raw* (*unaligned*) trends in yearly violent crimes in England and Wales for the PRC and CSEW from 2010/11–2022/23, as reported by [43]. Before 2014, the CSEW trend declines over time, while the PRC trend is flat. After 2014, the two trend lines diverge further, with police crime generally increasing over time and the CSEW survey estimates generally decreasing. They cross in 2017/18 when the amount of violent crime is found to be the same in both data sources. In 2022/23, there are 2,113,383 police recorded crime violent crimes which is over twice as many as the estimated 998,038 violent crimes in the CSEW. Note that due to the suspension of the face-to-face CSEW due to the COVID pandemic, data is not available between the year ending March 2020 and the year ending March 2022

### Seven possible reasons for the differences

We identify seven possible reasons for the differences between the measures of violent crime in Police Recorded Crime and the Crime Survey for England and Wales. Some of these reasons have been suggested by other researchers; and we have augmented this list with additional reasons of our own. We offer an initial assessment using logic and literature.

**1. There are differences between the survey and police because they use different temporal units.**

Flatley [45] suggested that the different time periods used for the CSEW and PRC may account for differences. While the police record crimes at the point of reporting, the CSEW records crimes in the 12 months prior to the interview. If violent crime is falling, then the CSEW will overestimate the amount of crime compared to police data; if violent crime is increasing then the CSEW will underestimate. However, this effect will be small as the difference will be no more than the change in the number of incidents from one year to the next, and therefore does not account for the large differences observed.

We conclude that any temporal effects would be minor.

**2. There are differences between the survey and police, including which violent crimes are included and different rules on counting and capping violent crimes when there is not one victim, one crime event, and one reporting event.**

The police data includes more forms of violence than the survey data. PRC includes all forms of violence criminalised in statute law. The CSEW does not include people who are killed (it is a survey of living victims), and the main module does not include some of the forms of violence only recently criminalised, such as harassment, or rarer violent crimes (such as kidnap) though it does have a special module on domestic abuse. Since the police data includes a wider range of forms of violence than in the main series from the CSEW, the total volume of crime might be expected to be higher. It is possible to identify the forms of violence common to both series, and we do this in the analysis below. It is possible to estimate the implications for the volume of crime if the full range of forms of violence (other than lethal violence) were included in the main module of the survey, and we do this in the analysis below.

The police data and survey data have different procedures to deal with various aspects of the complexity where there is not a simple one victim, one perpetrator, and one crime. When there are multiple crimes against the same victim, ‘caps’ have sometimes been introduced to limit the upper number. The CSEW caps the number of violent crimes against the same individual included in its estimates. High frequency *series crime* incidents, where the same crime is repeated against the same person in the same circumstances, are capped. The ONS justification has been to prevent large year to year variation in its time series. In the past, all *series crime* types were capped at five; the recent ONS crime series have capped at the slightly higher level of the 98^th^ percentile. This new cap, which varies by crime type, tends to lie between 8 crimes and 12 crimes for violence, deflating those reporting repeated crime above 12 crimes in a year. For example, for those in the upper 2% percentile, if they had suffered a violent attack once a week, the reported figure would have been 52, but in its estimates, the ONS would cap the figure at 12. Caps are relevant for repeated violent crime, such as domestic violent crime, and hence for violence against women. For example, Walby, Towers and Francis [4] found that the estimate of violent crime increased by 60% and the estimate of domestic violent crime increased by 70% when the cap of 5 was removed, when using CSEW data from 2011/12. Other ONS processing practices include not using the full calibrated weights where they are “too large”, insteadcapping them to a lower value. This particularly affects large households, who are therefore underrepresented in population estimates. Further issues concern the number of distinct crime reports that victims are allowed to complete- currently there is a maximum of six, which limits the information from highly victimised individuals. ONS estimated that changing the cap from 5 to the 98^th^ percentile increased the violent crime counts by only around 15% in 2016/17 [46]. Recalling that Walby, Towers and Francis [4] found a 60% increase when moving to the cap of 5 to uncapped crimes, use of the 98^th^ percentile cap by ONS means that there is still a substantial amount of surveyed crime that has been removed by capping.

Capping might appear to explain part of the discrepancy between PRC and the CSEW estimates, except that PRC is itself capped, though in a different way. The recording rule, known as the “Finished Incident Rule”, states that “*an incident comprising a sequence of crimes between the same offender (or group of offenders) and the same victim should be counted as one crime if reported to the police all at once*”. So, if a victim of domestic violence reports to the police that they have been hit repeatedly twice a week over the last three months, only one crime is recorded. However, if the victim reports each violent attack at the time it happens, then 26 crimes would be recorded. The number of violent attacks thus depend on the reporting behaviour of the victim. This is unsatisfactory for measuring crime incidents. Although we conclude that we can make no determination as to whether capping affects the Crime Survey more than PRC, nonetheless, the implications of the continued use of capping by both sets of estimates deserves further critical attention.

**3. There are differences between the survey and police because they refer to different populations by design.**

The PRC and CSEW refer to different populations of victims. The PRC include data on crimes against anyone, but they need to be reported to the police, while the CSEW includes data on crimes against only those included in its sampling frame and who choose to disclose it. While the PRC has no restrictions on which victims are included, the CSEW has restrictions because it is a sample survey. Walby and Allen [47] report on the limitations of the sampling frame of the CSEW (then BCS), which is restricted to those settled in their own homes. This excludes those staying temporarily with family and friends, and those in non-residential accommodation, such as refuges, hostels, care homes, student accommodation, long stay hotels, hospitals and prisons. Those excluded in this way are a more marginalised and disadvantaged group than the average population; and might be expected to be more likely to be victims of violence than the average person. The adult CSEW also excludes respondents only aged 15 or under in residences in England and Wales.

While we cannot estimate the effect of excluding those not in settled residential accommodation with the data available here, this is a task for the future. However, the scale of the impact of excluding children can be examined. We do this in the analysis section below. The CSEW has a greater tendency than PRC to exclude marginalised and minoritised people because of the limitations of its sampling frame. However, an estimate of the size of the effect of the remaining parts of the survey design (that is, only surveying households) on the amount of violent crime cannot be made with the data available here. This is a task for the future. There is a proposal [48] to incorporate a high-crime area booster sample into the CSEW to examine those most at risk in greater depth, which would ameliorate issues of small numbers but not solve the problem of the design of the sampling frame itself.

**4. Violent crime reported in the CSEW is declining because the response rate of the survey is declining and increasingly omitting the types of people who are most likely to be subjected to violent crime.**

**Fig. 3:**
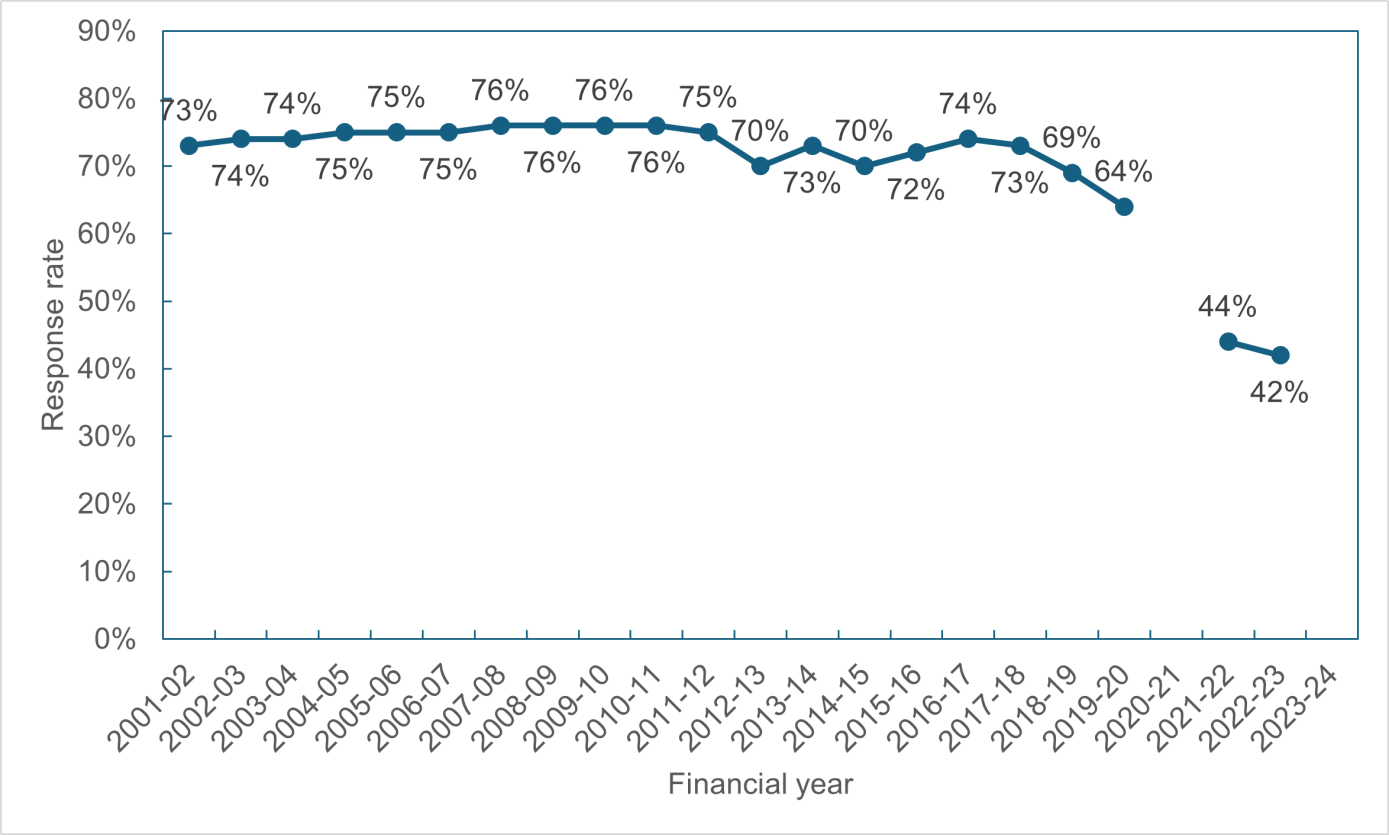
Response rates for the CSEW 2001-22 to 2022-23.

The response rate to the CSEW has fallen massively, with potential implications of leaving out the most victimised people. Fig 3 presents the response rates over the study period [48]. Before 2017/18, the response rate was relatively stable around 73%. After 2017/18, the response rate declines, to 64% in 2019/20 pre-pandemic, and to 42% in 2022/23. Could this have been the reason why there is such a large discrepancy between PRC and crime survey violence?

Are those responding to the survey a random sample of the population? Are the non-responders more likely to be in situations of vulnerability to violence than responders? As Hope [49] says, “the kinds of people who refuse or cannot be contacted are precisely the kinds of people who are most likely to be vulnerable to crime victimization.” Verian and ONS [50] give the response rates for socio-demographic subsets of the 2022/23 sample. Response rates are particularly low for the area classification labelled as “Ethnicity central” (30%), with high proportions of non-contact and refusals. This classification is defined by ONS as “predominately located in the denser central areas of London”, with all non-white ethnic groups having a higher representation than the UK average [51]. In terms of region, London had the lowest response rate (32%), with a particularly high non-contact rate of 23% among those not responding. Many of this group are likely to be the more disadvantaged and marginalised in society with their consequent high victimisation rates. However, they may call the police if violently victimised.

Decreasing response rates to national surveys are not unique to the CSEW. The Labour Force Survey (LFS) response rate declined to 14.6%, June to August 2023, leading to the partial cancellation of the ONS’s labour market data release on the grounds its lack of reliability [52]. The response of the ONS to the low response rate in the CSEW is different to that for the LFS. In the recent data quality review of the crime survey, ONS [53] stated that the quality of the CSEW data for 2022–23 was broadly similar to CSEW data for 2019–20, when they were accredited official statistics, and was not substantially affected by the lower response rate. They base this assessment on their interpretation of the work by Sturgis et al. [54] who investigated the relationship between non-response rates and non-response bias. However, the Sturgis et al. analysis compared those answering the survey at the first door-knock to those who answered eventually at later contacts, treating the latter group as non-responders. The group of “never-responders”- those who never answer or those with complete non-contact - were never investigated by them. It is, however, the never-responders which provide the non-response in the crime survey as the survey design allows for repeated calls to be made to an address. The ONS’s use of the Sturgis et al paper as evidence that low response rates are unproblematic is therefore not correct.

Are the non-responders different to the responders? ONS uses survey weights to allow the responders, once weighted, to represent the non-responders based on factors including the Census Output Area Classification, and the 12-category ONS urbanity classification. However, there is evidence to suggest that *unmeasured* variables such as health and language issues are factors discouraging residents from answering the door, especially in high crime areas [55,56]. We suggest that other unmeasured variables such as previous negative experiences with officialdom, mistrust and involvement in low-level crime may also be factors in refusal to respond to the door-knock.

In contrast to ONS, we are concerned that the low response rate achieved in 2022–23 may well account for a substantial part of the drop in CSEW violent crime between 2019–20 and 2022–23.

In conclusion, the recent low response rates mean that the CSEW has a growing tendency to exclude violent crime against marginalised and minoritised people, thus widening the gap between police recorded crime and CSEW reported crime.

**5. The disclosure of domestic violence to the CSEW varies between the main questionnaire and the specialised self-completion module, raising the question as to whether domestic violence and other forms of violence are under-estimated in main CSEW data series.**

The fifth issue concerns the methodology that affects disclosure to a survey. There is a contrast in lower disclosure rates between the main module where questions are asked face-to-face (F2F) and the higher disclosure rates in specialised domestic abuse module where questions are answered by self-completion (SC). The self-complete module surveys the same respondent, but uses computer assisted self-interview (CASI) to allow for greater confidentiality in the survey responses on domestic violence

An important point is that the self-complete part of the CSEW measures domestic abuse, which is broader than the domestic violence definition used in the face to face module. Domestic violence is physical abuse, whereas domestic abuse includes threats, sexual assault and stalking as well as force (domestic violence). The self-complete part of the CSEW produces a greater estimate of the number of victims of domestic violence (prevalence). Walby and Allen [47], using 2001/2 data, estimated that 12-month prevalence rates for domestic violence among 16–59 year-olds were five times greater in the self-complete (SC) module than in the face-to-face (F2F) module. Walby, Towers and Francis [4], using 2011/12 data, found a ratio of 3.8, whereas ONS [57] found a ratio of 8.0 from the 2016/17 survey. Most recently, Cooper and Obolenskaya [58] found a ratio of 2.1.

What are the reasons for this difference? as recent ONS report [57] states that the self-complete module “*allows respondents to feel more at ease when answering these sensitive questions, due to increased confidence in the privacy and confidentiality of the survey*”. There is both increased confidentiality for the respondent, with not even the interviewer knowing the answers entered into the computer, and also different wording, for example, the word “crime” is not used.

There are some limitations, however, since in comparison to the F2F survey, there is poor information on the number of violent attacks experienced, the module is not administered to 10–15 year olds, and has only recently been offered to those over 65.

There are difficulties in measuring *crimes* of domestic violence in the self-complete survey, which is focused on victims rather than events. The question on the *frequency* of assaults (crimes) rather than its prevalence (whether it happened at all to a victim) is asked only sporadically in the self-complete module and is a categorical response rather than a numerical response. However, researchers have made approximate estimates of the number of domestic violent crime incidents per victim using self-complete data to compare with estimates using the F2F module. Walby, Towers and Frqancis [4] found that the frequency of domestic violence crimes per victim was 1.75 times greater in the self-completion module than the face-to-face module in 2008/9. We then combine the ratios of increased numbers of victims of domestic violence with the ratios of the increased number of crimes of domestic violence for each victim. This gives an overall SC/F2F ratio for violent crime *incidents* of 6.6 (1.75 x 3.8). Using the prevalence ratio of 2.1 Cooper and Obolenskaya [58] instead, gives an overall SC/F2F incidence ratio of 1.75 x 2.1, or 3.75. Estimated domestic violence crimes therefore increase between three and a half and six times when measured by the self-complete methodology as compared to the face-to-face methodology.

Given the success of the self-complete methodology in generating higher levels of disclosure of domestic violence, there is an issue as to whether this methodology might be used for asking all questions about violence, including from acquaintances and strangers. Experiences of violence are often sensitive and respondents may not wish to talk about crimes of violence in front of their household or their interviewer (and therefore less likely to disclose in the F2F module). Self-reporting to a computer avoids these problems. Further, there are innovations in wording about violence in the SC module. Experiences of violence by customer-facing staff (acquaintance or stranger violence) may not be viewed by the victim as criminal, so applying these innovations in wording could also be considered. However, it would also be important to keep the innovation of the victim form, in order to ensure adequate information on the frequency of crimes as well as the number of victims.

The methodological differences between the F2F and SC contribute to the difference in volume between the PRC and CSEW, since only data from the F2F is used in the overall CSEW estimate of violent crime. Using self-complete methodology for all types of violence, while including the innovation of the victim form used in the F2F, would narrow the gap between the two series. We investigate this in the analysis section below.

**6. Violent crime reported by the police is increasing because more people who experience violent crime are reporting it to the police or having it reported to the police by other people.**

Hales [59] noted that since 2015, the PRC recording practice changed. Crimes reported by professional third parties needed to be recorded and counted, allowing medical professionals and social workers to report crime without verification from the victim. This will have led to some increases in the police recording of crimes against vulnerable people, such as child abuse and elder abuse, in the PRC, and possible some increases in the trend, as the new recording practice was adopted fully. This has probably only a small effect.

There may be more reporting of violence to the police by victim/survivors as a consequence of the greater normality of discussing these matters in popular culture. It is hard to estimate the scale of effects of such a change over time.

**7. Violent crime reported by the police is increasing because the police are getting better at recording the violent crime reported to them.**

There has been substantial concern about police under-recording of crime for over a decade from public interest groups, the government, and HMIC (as noted earlier). This has concerned crime in general, and violence against women in particular. In 2014, HMIC drew attention to the fact that only two thirds of violent crimes reported to the police in the CSEW being actually recorded by the police. Since 2014, pressure from HMIC and regular unannounced inspection of police forces on a rolling programme has improved accuracy.

#### Overall crime.

The May 2024 review of PRC [1] concluded that “*police forces have made significant improvements to crime recording, but there are common challenges to ensuring the quality of recorded crime data*”. The review examined 23 police forces and found an overall accuracy of 92%. This percentage is higher than the 80% recorded in 2014. Further progress in improving the accuracy of police crime recording will, in our view, be likely to make only minor changes to the PRC data series. The estimated increase for general crime is 15% (92/80) and for violent crime may be as large as 37% (92/67) but is not sufficient to fully explain the threefold increase in the PRC/ CSEW ratio from 2014–15–2022–23.

#### Domestic abuse.

HMIC were also concerned in 2012 about the poor processing and recording of domestic abuse cases by the police. They later reported [28] that overall, between 2012–13 and 2014–15, there has been a 31% increase in domestic abuse related crimes recorded by the police, which has been due in part to improved recording. We can examine later changes through the percentage of violent PRC cases which have a domestic abuse related flag. Table 1 [44] shows data from 2015–16, together with the *number* of violent police recorded crimes that were domestic abuse related.

**Table 1:** PRC violence flagged as domestic abuse 2015-16 to 2022- 2023, England and Wales.

Financial year	Violence against the person domestic abuse-related offences	Percentage of police recorded violence offences that were domestic abuse-related
2015-16	319,224	33.0%
2016-17	366,375	32.4%
2017-18	445,494	32.7%
2018-19	564,005	34.6%
2019-20	610,024	35.4%
2020-21	656,448	37.8%
2021-22	706,941	34.5%
2022-23	702,944	34.0%

Flagged domestic abuse is part of the increase in PRC from 2015 onwards. The *number* of domestic abuse cases that are ‘violence against the person’ increases between 2015/6 and 2021/2, a pattern similar to other PRC violence. However, while the number of cases of violence against the person flagged as domestic abuse related by the police has more than doubled in the period 2015–2023, the percentage of cases flagged as domestic abuse has shown far less change, with a small increase from 33% in 2015/6–38% in 2020/21, but then a decline to 34% in 2022/3. We conclude that while the HMIC domestic abuse initiative contributed to an increase in the proportion of violent crime recorded by the police and flagged as domestic, this initiative is not sufficient to account for the later divergence in the trends of the two series (PRC and CSEW) since the proportion of cases flagged as domestic-abuse related by the police changes only a little.

## Materials and methods

We are interested in improving the estimates of the trend in violent crime in the population over time and in the amount and severity of violent crime. We have identified challenges in this measurement and also opportunities to improve on existing estimates. We address four of these in the analysis below concerning violence against children (omitted from the main CSEW but included in a special module), comparing the trend in domestic violent crime in the main and specialised modules of the CSEW, improving the comparison of the trend between the two data sets by developing a smaller sub-set of forms of violence common to both data series, and improving the measurement of the volume of violent crime by applying the ratio of violence reported or not reported to the police disclosed to the CSEW back on the PRC data.

We use the two data sources, police recorded crime (PRC) and the Crime Survey for England and Wales (CSEW), as introduced in the earlier section.

For estimates of violence from the CSEW, we have mostly used published reports. This includes the estimated number of adult (16+) violent crimes in England and Wales by severity and year in Table A1 of an ONS report [43], together with the percentage reported to the police by severity and year [60] the estimated number of 10–15 child violent crimes in England and Wales by severity and year [61], estimated survey response rates reported by ONS [48], and CSEW self-complete domestic abuse estimates for all victims [44]. All published reports were accessed on 15/08/2024 and the weblinks checked on 22/10/2024 to ensure that the links were live. We have additionally analysed (a) the raw survey responses from the child surveys from 2010/11–2019/20 to obtain estimates of police reporting rates for children aged 10–15 and (b) the number of domestic violence incidents and victims from the anonymised face to face survey accessed 17/10/24) [62], which are not available in published reports. These yearly datasets are fully anonymised, with no identifying information.

## Analysis

### Children

One of the differences between the police/PRC and survey/CSEW data is that the police include crimes against children, while, usually, the CSEW does not since it is usually asked only of people aged 16 and over. Does the inclusion or exclusion of children as victims of violent crime make much difference to the trend and volume of violence? It is possible to investigate this since the CSEW has on occasion had a separate survey on children. We use this to investigate the scale of the implications of their inclusion or exclusion.

ONS provide estimated violence figures from the separate child Crime Survey in appendix tables to the CSEW up to 2020 [61]. For 2022–23, the relevant child crime incidence figures are not reported because of low response rates. Reporting rates to the police are not provided, but we have calculated them from the CSEW public survey datasets stored at the UK Data Service [61], using individual weights, and using the same definition of violence as the adult survey. Reporting rates are calculated by utilising the Child survey question CCOPSKNO “Did the police find out or know about what happened?”. Table 2 compares adult and child violent crime estimates, reporting rates, and estimated reported violent crime incidents from 2010–11–2019–20. We can observe that child violence is different in nature to adult violence.

**Table 2:** Adding data from the child CSEW survey: adult and child violent crimes, police reporting rates and estimated reported violent crimes.

Financial year	Adult crimes age 16 or more	Child crimes age 10 -15	Adult police reporting rate	Child police reporting rate	Adult police reported crimes	Child police reported crimes
2010-11	2,126,494	744,000	41.0%	13.3%	872,361	98,952
2011-12	1,956,485	697,000	43.4%	8.5%	849,434	59,245
2012-13	1,936,369	532,000	42.5%	7.3%	823,600	38,836
2013-14	1,562,228	454,000	54.3%	8.0%	848,967	36,320
2014-15	1,725,530	410,000	53.7%	4.1%	926,886	16,810
2015-16	1,467,984	550,000	52.9%	6.9%	776,001	37,950
2016-17	1,432,009	419,000	43.3%	7.8%	620,329	32,682
2017-18	1,426,150	423,000	39.4%	5.3%	561,724	22,419
2018-19	1,344,384	457,000	42.9%	7.8%	576,711	35,646
2019-20	1,238,572	298,000	46.5%	7.3%	575,933	21,754

There is more of it, with the extra six years of age providing on average an extra 30% of violent crimes on top of the adult crimes. However, very few of them are reported to the police. In general, after 2013/14, child reported violence will add on an additional 25,000–35,000 violent offences per year, or at most an additional 5–6% of offences.

Improving the sampling frame by including the child CSEW data will not increase police-reported CSEW crimes by more than 6%. This is a significant increase, but relatively small in comparison with the scale of the difference between the PRC and CSEW.

### Comparative disclosure rates in the F2F and SC of the CSEW

We examine the trend in CSEW self-complete (SC) domestic abuse, to contribute to a comparison of the methodology of the F2F and SC. The CSEW SC asks the same people in the same interview setting about a specific subset of the violence against them (domestic abuse) in the same period. The SC includes more crimes (non-physical abuse, stalking, threats and sexual assaults) and is focused on victims rather than crime incidents.

**Fig. 4:**
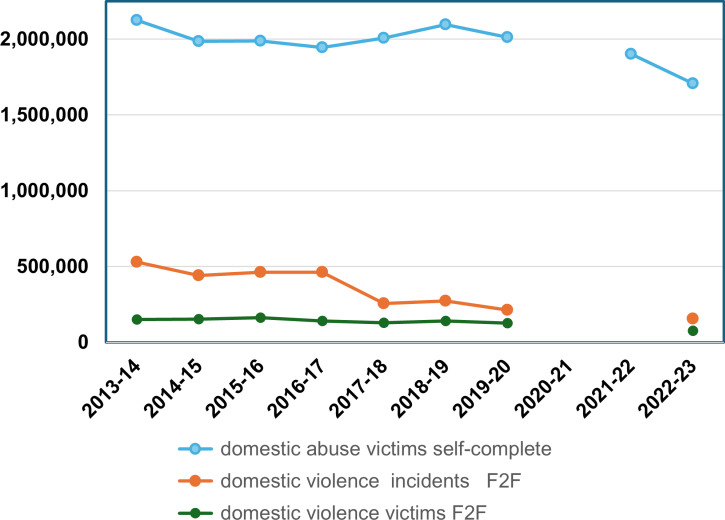
Estimated domestic abuse victims from the CSEW self-complete and domestic violence victims and incidents from the CSEW F2F, 2013-2023.

Fig 4 shows the trends in CSEW SC domestic abuse, using data from [44] compared with trends in CSEW F2F domestic violent crime (calculated by us from the raw survey data). The estimated numbers are shown in Table 3. This shows that in the period 2013–4–2019–20 the trend in the number of victims of domestic abuse in the CSEW SC is flat. It does not have the downward trend found in the CSEW F2F for incidents of domestic violent crime in the same period. In the most recent period, 2021/2–2022/3, however, it does show a decline, parallel to the F2F.

**Table 3:** Estimates of domestic abuse victims from the CSEW self-complete module and estimated victims and incidents from the CSEW F2F 2013-2013.

	Estimated domestic abuse victims self-complete	Estimated domestic violence incidents CSEW F2F	Estimated domestic violence victims CSEW F2F
2013-14	2,126,000	528,411	149,684
2014-15	1,985,000	439,687	153,478
2015-16	1,989,000	463,008	162,442
2016-17	1,946,000	462,497	140,408
2017-18	2,007,000	255,294	129,097
2018-19	2,096,000	272,168	141,428
2019-20	2,013,000	212,131	125,566
2020-21	NA	NA	NA
2021-22	1,902,000	NAA	N/A
2022-23	1,707,000	155568	76,848

Additionally, the overall estimate of domestic abuse victims and domestic abuse incidents in the self-complete CSEW is much larger than that of estimated victims and incidents of domestic violent crime in the main F2F module. Disclosure of violence is sensitive to the mode of delivery of the questionnaire.

### Re-estimating trends by creating an aligned subset of data

The CSEW and PRC differ in the types of violence that are included in their datasets. We seek a set of types of violence that is common to them both, in order to compare the impact of their methodology of data gathering. There are two differences. The CSEW includes only a subset of the types of violence included in the PRC. The PRC includes only crimes reported to the police. To compare the CSEW and PRC methods of measuring violent crime, we seek a shared, or aligned, list of violent crimes that can be found in both data series. This can be done be narrowing the data set to those crimes included in both. Or it can be done by estimating the implications of including the violent crimes excluded. We start with the first method for the analysis of trends, and turn to the second one later for the analysis of the volume of violent crime. For the first method of creating an aligned data set, this means

a) limiting the violent crimes in the PRC to the smaller set that is included in the CSEW
b) limiting the estimates of violent crime in the CSEW to those incidents that respondents tell the survey were reported to the police by themselves or others.

This method of alignment produces an under-estimate of the amount of violent crime, since it excludes the violence that is recorded in one dataset but not the other. This method of alignment is used to examine the trend. We return to the improvement of the estimate of the volume of violent crime later.

The first step in the alignment process forms a subset of violent crimes that is comparable across the two data sources. Table 4 shows the comparable subset of crimes for violence, and their categorisation into violence severity groups. We exclude from the police data the categories of violent crimes that are not included in the CSEW. We include CSEW violent crimes with a sexual motive as action rather than motivation defines a violent act. The violent offences that are excluded from the police data include murder, attempted murder, manslaughter, threat to kill, conspiracy to murder, cruelty to children, kidnapping, modern slavery, and all stalking and harassment offences. These crimes are not or cannot be

**Table 4:** Comparable subset of violent crimes in CSEW and police recorded violent crime offences, with severity categorisation and number of crimes in 2022/23.

Violence severity	CSEW offence and CSEW code	Estimated CSEW violent crimes in 2022/23	Police recorded Crime offence and Home Office code	Police recorded violent crimes 2022/23
Assault with injury	Serious wounding (11)	36,895	Assault with intent to cause serious harm (from 2012/13 on) (5D)	35,263
	Serious wounding with sexual motive (31)	0	Assault with injury (from 2012/13 on) (8N)	516,251
	Other wounding (12)	112,933	Racially or religiously aggravated assault with injury (from 2012/13 on) (8P)	3,795
	Other wounding with sexual motive (32)	8,211	Assault with injury on a constable (introduced in 2015/16) (8S)	11,022
			Assault with injury on an emergency worker (other than a constable) (from April 2020) (8T)	3,388
			Wounding (up to 2011/12) (5A)	0
			Actual bodily harm [ABH] (up to 2011/12) (8G)	0
			Racially or religiously aggravated ABH or other injury (up to 2011/12) (8J)	0
Assault without injury	Common assault (13)	678,413	Assault without injury on a constable (104)	29,308
	Attempted assault (19)	161,585	Assault without injury (105A)	684,349
			Racially or religiously aggravated assault without injury (105B	10,026
TOTAL		998,037		1,293,402

measured in any victimisation survey (such as fatal violence) or are rare crimes such as kidnapping. The naming and offence categorisation of offences in police recorded crime has changed over time, but the data series is reasonably consistent.

The second step in the alignment process limits the CSEW estimate to those violent crimes that respondents state have been reported to the police by themselves or others. The CSEW asks respondents whether the violence was known to the police and the ONS publishes data on this using the survey question COPSKNOW “Did the police come to know about the matter?”. [60] makes these percentages available for assault both with and without injury and applies these to the ONS raw estimates of violence [43].

Thus, we align the series by, first, using the (narrower) CSEW definition of violence for both data series and, second, restricting the CSEW estimates to those that were reported to or otherwise known to the police. This narrows the amount of violent crime included in the comparative analysis to that included in both data sets. This is thus an underestimate of the volume of violent crime, excluding ‘dark’ data that is in one or the other dataset but not in both datasets. The ‘aligned data’ in two data series now measure the same kind of reported violent crimes over time.

We compare the *aligned* CSEW (only those reported to the police) estimates with *aligned* police reported crime figures (using the narrow CSEW definition of violent crime) from 2010/11–2022/23. We report both on trends in overall violence and disaggregated by severity of violence. CSEW violence can be disaggregated by severity into serious wounding, other wounding (together making assault with injury), and common assault without injury [50].PRC data can only be disaggregated into violence with injury and violence without injury. We use the second and coarser of these two categorisations.

Table 5 shows the estimated CSEW violent crimes and the proportion of those that respondents tell the survey that were known to (reported to) the police, 2010–11–2022–23, disaggregated by severity of violence. On average, in 2022–23, 40.9% of violent crimes reported to the CSEW were reported to the police. The percentage reported to the police is slightly higher for assaults with injury (45.3%) than those for assault without injury (37.6%).

**Table 5:** CSEW number of violent crimes by whether reported to the police, 2010/1-2022/3.

Financial year	Assault with injury			Assault without injury			Total violence		
	Number of crimes	reported %	reported crimes	Number of crimes	reported %	reported crimes	crimes	reported %	reported crimes
2010-11	1,232,143	45.4%	559,802	894,351	34.9%	312,558	2,126,494	41.0%	872,361
2011-12	1,022,681	51.0%	521,299	933,803	35.1%	328,135	1,956,485	43.4%	849,434
2012-13	1,144,307	47.1%	539,518	792,062	35.9%	284,082	1,936,369	42.5%	823,600
2013-14	663,978	54.7%	363,103	898,250	54.1%	485,865	1,562,228	54.3%	848,967
2014-15	837,727	59.2%	495,892	887,804	48.5%	430,993	1,725,530	53.7%	926,886
2015-16	630,526	57.5%	362,666	837,458	49.4%	413,336	1,467,984	52.9%	776,001
2016-17	701,823	43.4%	304,428	730,186	43.3%	315,901	1,432,009	43.3%	620,329
2017-18	665,847	42.6%	283,808	760,303	36.6%	277,916	1,426,150	39.4%	561,724
2018-19	714,310	48.8%	348,887	630,074	36.2%	227,824	1,344,384	42.9%	576,711
2019-20	590,679	57.0%	336,517	647,893	37.0%	239,416	1,238,572	46.5%	575,933
2020-21	NA	NA	NA	NA	NA	NA	NA	NA	NA
2021-22	NA	NA	NA	NA	NA	NA	NA	NA	NA
2022-23	423,212	45.3%	191,543	574,826	37.6%	216,263	998,038	40.9%	407,806

Table 6 reports the estimates of PRC crimes for the subset of violence that is comparable to the CSEW, in total and disaggregated by severity of violence. The numbers have reduced from the full violence definition. For example, in 2022–23, the number of recorded violent crimes was 2,113,383; examining just the aligned subset gives 1,293,402 recorded violent crimes. These estimates can be directly compared to Table 5.

**Table 6:** Police reported crime (PRC) violent crimes in subset of violence aligned to the CSEW definition of violence.

Financial year	PRC assault with injury	PRC assault without injury	Total PRC aligned violence
2010-11	350,937	225,547	576,484
2011-12	321,688	222,453	544,141
2012-13	310,541	216,815	527,356
2013-14	321,034	229,816	550,850
2014-15	371,906	292,089	663,995
2015-16	429,722	365,215	794,937
2016-17	465,202	431,404	896,606
2017-18	510,236	534,070	1,044,306
2018-19	546,062	613,351	1,159,413
2019-20	537,516	653,329	1,190,845
2020-21	463,059	595,237	1,058,296
2021-22	563,241	712,390	1,275,631
2022-23	569,719	723,683	1,293,402

**Fig 5:**
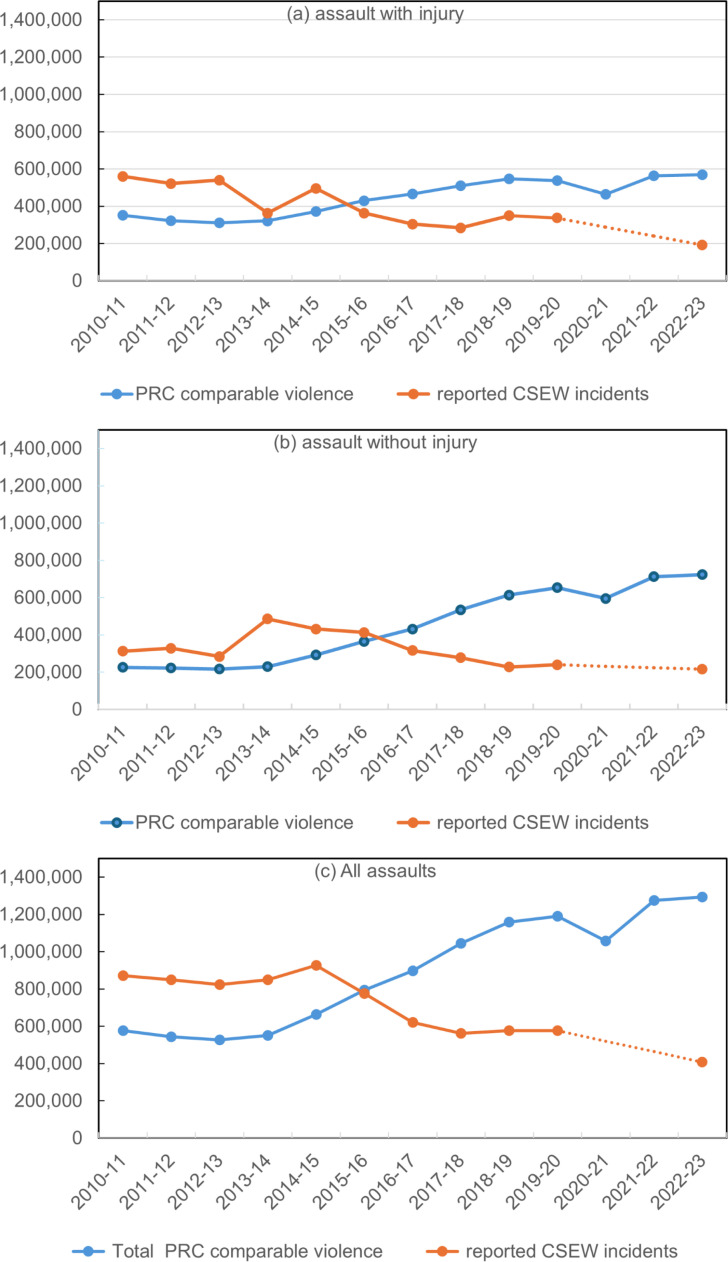
Trends in PRC and CSEW subsets of violent crime aligned for definition and reporting.

Fig 5 shows the data in Tables 4 and 5 in graphical form over time. It shows aligned PRC and CSEW violent crime for assaults, distinguished between assault with injury and assault without injury. This shows that, after aligning for different definitions of violence and for whether reported to the police, there is, largely, an upward trend in police data and a downward trend in survey data, though with some minor fluctuations and periods of stability.

For assaults with injury, there is a 77% increase in PRC from 2013/14 from 321,034 to 569,719 in 2022/23, with a small pandemic dip in 2020/21. In contrast, the estimated CSEW reported crime data shows a decline over the same period of 53%, from around 363,000 to 192,000. For assaults without injury, the changes are even larger. PRC more than trebles, from 229,816 to 723,683. CSEW reported crime, in contrast, more than halves, from 485,865 to 216,863. When we combine into overall assault, PRC shows an *increase* of 134% over the same time period from 550,850 to 1,293,492 and CSEW a *decrease* of just over half from 848,467 to 407,806.

Previous official reports on the comparisons between PRC and CSEW reported crime [30,57,63] have used the ratio of the two figures to examine change over time. Early ratios of less than 1.0 prior to 2014 were originally used to show that PRC is underreported compared to the CSEW. Table 7 shows the ratios on the new violence series, disaggregated by violence severity.

**Table 7:** Ratios of PRC aligned violent crime to CSEW violent crime.

Ratios of PNC to CSEW reported violence comparable subset.			
Financial year	Assault with injury	Assault without injury	Total assault
2010-11	0.63	0.72	0.66
2011-12	0.62	0.68	0.64
2012-13	0.58	0.76	0.64
2013-14	0.88	0.47	0.65
2014-15	0.75	0.68	0.72
2015-16	1.18	0.88	1.02
2016-17	1.53	1.37	1.45
2017-18	1.80	1.92	1.86
2018-19	1.57	2.69	2.01
2019-20	1.60	2.73	2.07
2020-21	NA	NA	NA
2021-22	NA	NA	NA
2022-23	2.97	3.35	3.17

The high ratios in later years challenge the assumption that the PRC is always lower than the CSEW. By 2016–17, all ratios are greater than 1.0. In 2022–23, assault without injury and total assault both have PRC/CSEW ratios over 3.0, with assault with injury very close to three. There is a very large increase in the ratio between 2019–20 and 2022–23. In the most recent year, on a data set aligned for the definition of violence and whether reported to the police, the PRC data shows more than three times the amount of violence than the CSEW.

### Estimating the overall level of violent crime for 2022/23

What is the overall level of violent crime, both reported and not reported? We use first the narrow, aligned definition of violent crime, and second, we estimate the implications of taking into account the crimes not reported to the police, and third, the implications of using the wider police (statutory) definition of violent crime.

First, we focus on the narrower aligned violent crime. The PRC aligned violent crimes in 2022/3 was 1,293,402, while the CSEW aligned violent is estimated to be 407,806 (see Table 5).

Second, we estimate the implications of including violence not reported to the police (using knowledge from the survey). The CSEW finds that 40.9% of violent crime reported to the CSEW is reported to the police (as reported by survey respondents to the CSEW) (see Table 5). Hence, in estimating the number of violent crimes in the population, the number of violent crimes recorded by the PRC should be increased by a factor of 100%/40.9%, or 2.445 (thus including those not reported to the police). On the aligned definition of violent crimes, the estimate of the number of violent crimes in England and Wales in 2022–23 would be 1,293,402 × 2.445, or 3,162,367 PRC crimes compared to 998,038 CSEW crimes for 2022–23.

Third, we estimate the implications of moving from the aligned definition to the wider police (statutory) definition of violent crime. The police reported 2,113,383 incidents of violent crime in 2022–23. The CSEW finds that 40.9% of violent crime reported to the CSEW is reported to the police. If we assume the same reporting rate for other forms of violent crime (except for homicide, and death or serious injury by unlawful driving, where we assume a 100% reporting rate), then the estimate of all violence against the person offences increases to 5,164,983. (See Table 8).

**Table 8:** Estimating violent crime from police recorded crime and rates of reporting to the police from the CSEW. England and Wales 2022/23.

Type of police recorded crime violence	Police recorded crime 2022/23	Estimated percentage of violent crime reported to the police	Estimate of violence against the person offences 2022/23 including those not reported.
Homicide	602	100%	602
Death or serious injury -unlawful driving	929	100%	929
Violence with injury	573,791	40.9%	1,402,912
Violence without injury	828,673	40.9%	2,026,095
Stalking and harassment	709,388	40.9%	1,734,445
**TOTAL**	**2,113,383**		**5,164,983**

On the basis of the police definition of violent crime, the data in the PRC in 2022/3, and data from the CSEW that 40.9% of most violent crime is reported to the police (except for 100% for homicide and death or serious injury from unlawful driving), the number of violent crimes is 5,164,983.

This is more than double the number of police recorded violent crimes, of 2,113,383. It is more than five times the number of CSEW violent crimes of 998,038. The CSEW provides a serious underestimate of the volume of violent crime in England and Wales today.

## Discussion

The differences in the volume and trends in violent crime provided by the CSEW and PRC are very large. Seven potential methodological reasons for differences between the CSEW and PRC have been investigated. We find:

1. There are differences between the survey (CSEW) and police (PRC) because they use different temporal units. However, these differences are small and are likely to have very little effect.
2. There are differences between the survey and police because the survey has a narrower definition of violent crimes than the police. The survey excludes homicide, death by dangerous driving, and the main module does not include some forms of violence that have recently been made criminal offences, such as harassment. This significantly reduces the volume of crime reported in the survey as compared with the police.
3. While both the survey and police have complex rules on capping, counting and prioritising violent crimes when there is not one victim, one crime event, and one reporting event, which reduce the volume of violent crime against the most victimised, they do so in different ways. This probably significantly reduces the violent crimes measured by the police, but it is hard to estimate the size of the difference and how it has changed over time.
4. There are differences between the survey and police because they refer to different populations. The survey population is narrower than the police population since the sampling frame limited to those aged 16 or over, living in settled, residential households, while the police data as no such limitations, for example, including children under 16, the homeless, and those living in hostels, or staying with family and friends. The exclusion of the most marginalized people by the CSEW sampling frame probably substantially reduces the amount violent crime reported, but it is probably stable, though hard to estimate the exact amount.
5. The recent decline in the response rate probably disproportionately excludes further people vulnerable to violence among the more marginalised and minoritised and may also have effects on the trend reported. The decline in the response rate probably disproportionately omits people in the types of situations of vulnerability who are most likely to be subjected to violent crime. However, the debate on the extent of the impact of the decline in response rate on the inclusion of people at high risk of violence is not fully resolved.
6. The main survey module of the CSEW has lower rates of disclosure than the self-completion module for comparable crimes, suggesting that there is significant under-disclosure in the main module. There are different rates of disclosure for comparable domestic violent crimes between the main module and self-completion modules of the CSEW, with much higher rates of disclosure of domestic violence in the self-completion module than in the main module which is asked face to face. The significantly lower disclosure rate in the main module than in the self-completion module for those forms of violent crime that are comparable suggests that the real rate violent crime in the population may also be significantly higher than is reported in the main module. This is probably stable over time.
7. Violent crime reported by the police is increasing because more people who experience violent crime are having it reported to the police, as a result of the police including unverified third-party reporting, though this is probably relatively small in overall effect. Violent crime may be increasingly reported to the police by victim/survivors. This may apply particularly to domestic and sexual violence as a result of greater normality of discussing these crimes in popular culture.
8. Violent crime reported by the police is increasing because the police are getting better at recording the violent crime reported to them. There has been substantial pressure by public interest groups on the police to improve their activities in relation to violence against women, sexual violence, and domestic abuse, reported by the media, and articulated in repeated investigations by the HMIC. The timing of this pressure correlates with an increased recording of violent crime, suggesting a connection between the pressure and the improvement.

## Conclusions

We draw nine conclusions about methodology and implications for the volume and trend in violent crime.

First, there is substantially more violence than previously estimated. When data from the CSEW on the rate of reporting of violent crime to the police (40.9%) is combined with PRC data on violent crimes recorded by the police, the estimate of the volume of violent crime (5,164,983) is over five times higher than the estimate from the CSEW (998,038) that is treated as the preferred official statistic. Dark figures of crime exist in both data sources and need to be understood.

Second, the estimate of the trend in violent crime is uncertain because of the scale of the effects of the methodological issues. The previous consensus that violent crime is declining needs to be reconsidered.

Third, the CSEW has always underestimated violent crime, and this is becoming visible now that the quality of the police data (PRC) has improved.

Fourth, the police (PRC) have a better estimate of volume of violent crime than the survey (CSEW) – the police estimate being three times higher than the survey estimate once the series are aligned– but this is still an underestimate, since not all violent crimes are reported to and recorded by the police.

Fifth, the change in the ratio of violent crime reported by the survey (CSEW) and police (PRC) is the outcome of changes in the quality of the two data sources, a decline in the quality of the CSEW and a large improvement in PRC.

We draw further conclusions as to the implications of the substantive findings on the extent and trend in violent crime for the development of theory and policy.

Sixth, there are implications for criminological theory in its new account of the distribution of violent crime, and for social theory of inequality in its account of the greater amount of violence among the most marginal and minoritized. There is more violence than previously estimated, and this is likely to be disproportionately concentrated among those who are so marginalised and minoritised that their experiences are relatively excluded from the statistics generated by official surveys. The experiences of violence of the most marginalised and minoritised are underestimated in the data that is used to test theories of crime. The marginalised and minoritised are disproportionately victims of violent crime to a greater extent than previously recognised. The theorisation of inequality needs to take account of the fact that Britian is a more violent country than previously thought.

Seventh, there are implications for policy on official statistics to reach official standards on equality, diversity and inclusion, and for policy on the social protection of the marginalised and minoritised. In order to remove bias against the marginalised and minoritised in official statistics, there needs to be improvement in the quality of the CSEW to better include the experiences of the most marginalised and minoritised. The greater prevalence of violent crime has implications for policy on the relationship between marginalisation, minoritisation, and violent crime.

The implications for the ONS are that police data is now the better estimate of the volume of violent crime despite being limited to recorded crime, though it does not provide much information on its distribution in the population, and the changes in recording practices mean that the data on trends are not entirely reliable. As a step towards improvement, ONS should return to presenting the two series, CSEW and PRC, side by side, including both the F2F and SC variants, and discuss the relative strengths and weaknesses of each series for estimating the volume of violent crime, its distribution in the population, and for understanding its trends. Research needs to be commissioned to investigate the discrepancy between the two series in more detail.
